# *Vaccinia virus* Transmission through Experimentally Contaminated Milk Using a Murine Model

**DOI:** 10.1371/journal.pone.0127350

**Published:** 2015-05-22

**Authors:** Izabelle Silva Rehfeld, Maria Isabel Maldonado Coelho Guedes, Ana Luiza Soares Fraiha, Aristóteles Gomes Costa, Ana Carolina Diniz Matos, Aparecida Tatiane Lino Fiúza, Zélia Inês Portela Lobato

**Affiliations:** 1 Laboratório de Pesquisa em Virologia Animal (LPVA), Departamento de Medicina Veterinária Preventiva, Escola de Veterinária, Universidade Federal de Minas Gerais, Belo Horizonte, Minas Gerais, Brazil; 2 Laboratório de Patologia Veterinária, Departamento de Clínica e Cirurgia Veterinárias, Escola de Veterinária, Universidade Federal de Minas Gerais, Belo Horizonte, Minas Gerais, Brazil; Rega Institute for Medical Research, BELGIUM

## Abstract

Bovine vaccinia (BV) is a zoonosis caused by *Vaccinia virus* (VACV), which affects dairy cattle and humans. Previous studies have detected the presence of viable virus particles in bovine milk samples naturally and experimentally contaminated with VACV. However, it is not known whether milk contaminated with VACV could be a route of viral transmission. However, anti-*Orthopoxvirus* antibodies were detected in humans from BV endemic areas, whom had no contact with affected cows, which suggest that other VACV transmission routes are possible, such as consumption of contaminated milk and dairy products. Therefore, it is important to study the possibility of VACV transmission by contaminated milk. This study aimed to examine VACV transmission, pathogenesis and shedding in mice orally inoculated with experimentally contaminated milk. Thirty mice were orally inoculated with milk containing 10^7^ PFU/ml of VACV, and ten mice were orally inoculated with uncontaminated milk. Clinical examinations were performed for 30 consecutive days, and fecal samples and oral swabs (OSs) were collected every other day. Mice were euthanized on predetermined days, and tissue and blood samples were collected. Nested-PCR, plaque reduction neutralization test (PRNT), viral isolation, histopathology, and immunohistochemistry (IHC) methods were performed on the collected samples. No clinical changes were observed in the animals. Viral DNA was detected in feces, blood, OSs and tissues, at least in one of the times tested. The lungs displayed moderate to severe interstitial lymphohistiocytic infiltrates, and only the heart, tonsils, tongue, and stomach did not show immunostaining at the IHC analysis. Neutralizing antibodies were detected at the 20^th^ and 30^th^ days post infection in 50% of infected mice. The results revealed that VACV contaminated milk could be a route of viral transmission in mice experimentally infected, showing systemic distribution and shedding through feces and oral mucosa, albeit without exhibiting any clinical signs.

## Introduction

*Vaccinia virus* (VACV), an *Orthopoxvirus*, is the causative agent of bovine vaccinia (BV), which is an occupational zoonosis that affects lactating cows, their calves, and milkers. Cases of the disease have been frequently reported in several regions of Brazil since the end of the 1990s. Lesions begin with the formation of vesicles and papules that progress to ulcers and crusts with subsequent healing. A sudden decrease in milk production, mastitis, milkers’ absenteeism from work, medical expenses, and diseased calves are noted as the main losses caused by BV. The natural reservoirs of VACV have not yet been established. However, virus detection in sentinel and wild rodents suggests that they are the main reservoirs [1–3], as reported for *Cowpox virus* (CPXV) in Europe [4].

The Southeast is noteworthy among the Brazilian regions affected by BV, given the increasing number of outbreaks recorded and because it is the leading milk-producing region of Brazil. Milking and milk trading are known to proceed normally most of the time during BV outbreaks, despite herd involvement and the possibility of VACV transmission through milk and its derivatives. Both the VACV DNA and infectious particles have been detected in milk samples derived from cows naturally and experimentally infected [5–6]. Furthermore, viable viral particles have been isolated from experimentally contaminated milk and subjected to heat treatment [7].

The actual prevalence of VACV contaminated milk, derived from infected cows and the risk of infection by consuming contaminated milk and dairy products are still unknown. A study of experimentally infected lactating cows demonstrated that VACV DNA is present in milk up to 25 days post-infection (d.p.i), when the teats lesions have already healed [6]. These findings indicate that VACV may be present in milk even after the resolution of clinical signs of the disease. Therefore, there is a risk of transmission, as a cow without lesions would be considered healthy, and the milk would be used again for consumption and the production of dairy products.

Epidemiological surveillance is insufficient to control the disease, and the number of cases is most likely underestimated, despite the impact of BV outbreaks. The risk of infection in both humans and calves that ingest contaminated milk exists, because infectious viral particles have already been detected in the milk from naturally infected animals [5]. In Brazil, OPXV seroprevalence and possible exposure factors related to VACV infections were determined in humans who lived in regions that had outbreaks of BV [8]. Interestingly, OPXV neutralizing antibodies were detected in subjects of the study whom had no contact with BV affected cattle, and many of them had the habit of consumption of unpasteurized milk or artisan cheese, made with raw milk [8]. Although a direct cause-effect relationship could not had been established in that study, those findings suggest that consumption of VACV contaminated milk and dairy products may be a possible route for viral infection. Furthermore, Guray and collaborators [9], describing a *Buffalopox virus* (BPXV) outbreak in India with 166 human cases, reported that 17 (10.2%) cases had BPXV-related lesions in the mouth. The authors suggested that the mouth lesions could be due to raw milk consumption [9].

Therefore, the study of the possible transmission of VACV using contaminated milk becomes important, given the abovementioned reports. The murine model was chosen in the present study due to the known mice susceptibility to VACV infection, as has been shown by many experiments [10–13]. Furthermore, mice are ease to be handled and maintained. Therefore, this study aimed to examine VACV transmission, pathogenesis and shedding in mice orally inoculated with experimentally contaminated milk.

## Materials and Methods

### Cells

Vero cells (ATCC-CCL-81) were used for viral multiplication and plaque-reduction neutralization test (PRNT). BSC-40 cells (ATCC-CRL-2761) were used to detect the viable viral particles. Both cells lines were cultured using Eagle’s minimal essential medium (EMEM; GIBCO, USA) supplemented with 5% fetal bovine serum (FBS), gentamicin (50 μg/ml), potassium penicillin (200 U/ml), and Fungizone (GIBCO, USA) (2.5 μg/ml), under incubation at 37°C, in an atmosphere of 5% CO_2_.

### VACV-GP2 virus

The strain used to infect the milk was *Vaccinia virus* Guarani P2 (VACV-GP2), which was isolated from teat lesions of a cow during a BV outbreak that occurred in the municipality of Guarani, Minas Gerais (MG) State, Brazil, in 2001; the strain was serologically and molecularly characterized as VACV [14]. Viral purification was performed according to the method reported by Joklik [15]. Assays were conducted to assess the titer of viral stocks in six-well plates, according to the method reported by Campos & Kroon [16]. The titer was expressed as plaque-forming units per milliliter (PFU/ml).

### Milk samples and their contamination

The milk used in the experiment was obtained from Girolando cows, a breed of dairy cattle created in Brazil by crossing Gir cattle, a *Bos indicus* breed, with Holstein (*Bos taurus*) cows. All cows were serologically negative for *Orthopoxvirus* and belonged to the experimental farm of Veterinary School of Universidade Federal de Minas Gerais (UFMG). Five milliliters of milk was contaminated with VACV-GP2 containing 10^7^ PFU/ml in the final solution.

### Animal Ethics Committee Approval

The present study was approved by the Animal Ethics Committee (“Comitê de Ética no Uso Animal”, CEUA), of Universidade Federal de Minas Gerais (UFMG), Brazil, protocol 83/2011.

### Inoculation, monitoring, and sample collection

Forty BALB/c female mice, four-week-old, seronegative for *Orthopoxvirus*, were used. Ten of these animals received orally 100 μl of milk not contaminated with VACV (control group—CG), and thirty were orally inoculated with 100 μl of contaminated milk with 10^7^ PFU/ml (infected group—IG). Therefore, each mice from the infected group received an inoculum of 10^6^ PFU/100 μl of VACV contaminated milk.

The animals were kept in appropriate cages with individualized airflow for 30 days, and 10 animals were placed in each cage.

Two animals randomly selected from each cage were euthanized at 2, 5, 10, 20, and 30 days post-inoculation (that is, two animals from the control group and six animals from the infected group) to study the pathogenesis and distribution of the virus through the mice organs. The animals were euthanized according to CEUA recommendations, using 2% lidocaine in combination with 10 mg/ml thiopental at doses three times higher than the anesthetic dose, on each day of euthanasia. Samples of liver, tonsils, spleen, kidney, bladder, submandibular lymph nodes, tongue, heart, lungs, stomach, ileum, and cecum were collected during necropsy. Half of each organ sample collected was stored in buffered 10% formalin for histopathological examinations and immunohistochemistry (IHC) analysis, and the other half (without any preservatives) was used for PCR analysis and research of viable viral particles. The submandibular lymph nodes and bladder were only analyzed by histopathology and IHC because they are very small tissues, and the final volume obtained was insufficient to process them through PCR.

The mice were examined and weighed daily to observe the onset of clinical signs. Blood samples were collected from all animals prior to inoculation (T0) and at the time periods T2, T5, T10, T20, and T30, when the mice were euthanized. The blood collection was performed through the caudal vein using pre-sedation with xylazine (1 mg/kg) and ketamine (100 mg/kg) administered intraperitoneally. Serum samples were submitted to PRNT and the immunoperoxidase monolayer assay (IPMA), whereas the blood clot was diluted 1:10 to perform the nested PCR. The PCR positive samples were tested in a BSC-40 cell monolayer to observe the presence of viable viral particles.

Fecal samples were collected every other day using microcentrifuge tubes, placed directly on the anus of the animals. These samples were diluted 1:10 to perform the nested PCR [8–9], and the positive samples were submitted to virus isolation, in order to detect viable viral particles. A pool of the fecal, blood, and tissue samples for each collection day was performed per cage, thereby preparing one pool of samples from the control mice and three pools of samples from the infected mice per collection time. Oral swabs (OSs) were also collected every other day, and nested PCR was performed to assess the presence of viral DNA.

### Nested PCR for *vgf* gene amplification

Nested PCR assay targeting the growth factor gene (*vgf*) of the genus *Orthopoxvirus* (OPV) was used to detect the viral DNA in the samples collected from the mice, including feces, OSs, and tissues, as previously described [17]. As positive control, 10^5^ PFU/μl of purified *Vaccinia virus* Western Reserve (VACV-WR) were used. The same volume of samples added to each tube was replaced by water in the negative controls. The amplified fragments were fractionated by 8% polyacrylamide gel (PAGE) electrophoresis under 100 V voltage and silver stained.

### Virus isolation

PCR positive samples of feces, blood and Oss were inoculated into BSC-40 cells monolayers in six-well plates with 90% confluence for detection of viable viral particles. Three consecutive cells passages with 72 hours of incubation at 37°C were performed [18].

### Histopathology and immunohistochemistry (IHC)

Tissues fixed in 10% formalin were processed and embedded in paraffin. The blocks were sectioned into 5- to 6-μm slices in a standard rotary microtome and placed on a slide for hematoxylin and eosin staining (H&E).

Anti-VACV primary rabbit polyclonal antibody was used in the IHC at a 1:1000 dilution. The secondary and tertiary antibodies (peroxidase conjugated streptavidin) used were those from the Kit DAKO LSAB + System-HRP (Dako, USA). Two percent bovine serum albumin (BSA) was used for blocking. Ten percent hydrogen peroxide was used to block the endogenous peroxidase. The chromogen used was 3-Amino-9-ethylcarbazole (AEC; Sigma, USA). A teat section from a cow with lesions caused by VACV and lung tissue of mice inoculated intranasally with VACV were used as positive controls. The lung slides were kindly provided by the “Laboratório de Vírus, Instituto de Ciências Biológicas, Universidade Federal de Minas Gerais (UFMG)”. Tissues from the mice in the control group were used as negative controls.

Two scientists read the H&E and IHC stained slides independently. A qualitative analysis of the IHC positive slides was made by giving the average intensity score of 1+, 2+, or 3+, corresponding to focal, multifocal, and diffuse VACV-immunostaining, respectively.

### Plaque reduction neutralization test (PRNT)

Serum samples collected from the mice were subjected to the PRNT to detect anti-VACV neutralizing antibodies. The serum titer was calculated as the inverse of the highest dilution capable of neutralizing 50% of the number of plaques detected in the virus controls wells. Titers greater than or equal to 1/20 were considered positive [10].

### Statistical analysis

Bonferroni’s parametric test was used to analyze the animal weights for *p<0*.*05*, using GraphPad Prism Software, California, USA.

It is noteworthy that the number of animals used in the control group (n = 10), was lower (representing 25% of all animals) for ethical reasons, as recommended by the Animal Ethics Committee (“Comitê de Ética no Uso Animal”, CEUA), of Universidade Federal de Minas Gerais (UFMG), Brazil. The number of animals used was the minimum necessary to allow the statistical analysis of the comparison between control and infected groups.

## Results

### Clinical signs

No clinical signs were observed in the mice during the 30-day monitoring period, following the inoculation with VACV-GP2–contaminated milk. No significant difference was found between the groups when comparing the non-infected animals (CG) with the infected animals (IG) at each weighing time.

### Nested PCR and research of viable viral particles

#### Oral swabs

The nested-PCR analysis of the OSs showed that viral DNA amplification occurred intermittently between the 2^nd^ and the 10^th^ d.p.i. in at least one animal from each pool. The positive samples were inoculated into BSC-40 cells, and no lysis plaques were observed.

#### Feces and blood

The VACV shedding through feces was detected in the 6^th^, 8^th^, and 30^th^ d.p.i. Amplification of the *vgf* gene was observed in blood samples collected on euthanasia days (2^nd^, 5^th^, 10^th^, 20^th^, and 30^th^ d.p.i.) from the 5^th^ d.p.i. and at all collection times. Positive samples were inoculated in BSC-40 cells, and no lysis plaques were observed. The samples collected from the control group (CG) were all negative.

#### Tissues

The VACV DNA was amplified from lung, heart, tongue, kidney, tonsils, liver, and spleen samples in at least one pool analyzed at the different collection times. The tissue samples from the CG were PCR-negative. The positive samples were inoculated in BSC-40 cells, and no lysis plaques were observed.

### Histopathology and immunohistochemistry

**Fig 1:**
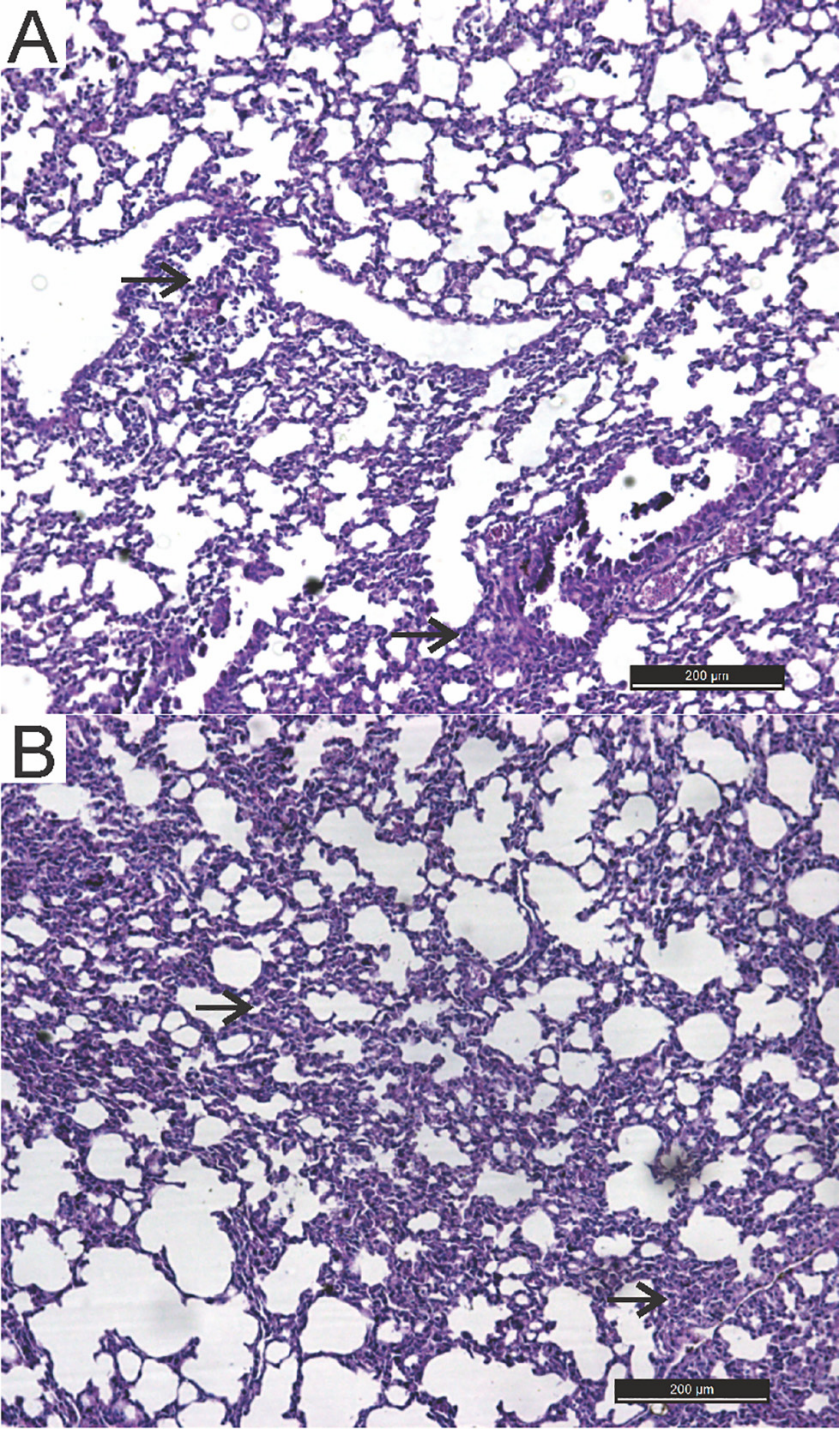
Histological lung sections showing interstitial lymphohistiocytic infiltrate (arrows). H&E staining. (A)- Mouse lung from the control group (CG) inoculated orally with milk not contaminated with VACV (200 μm). (B)-Mouse lung from the inoculated group (IG) orally inoculated with VACV-contaminated milk (200 μm).

A portion of the tissues collected from the euthanized mice was submitted to histopathology and IHC methods. The lungs of all inoculated animals displayed moderate to intense lymphohistiocytic infiltrates in the interstitial space, which is characteristic of interstitial pneumonia, upon histopathology examination. The control group animals showed mild interstitial pneumonia, most likely resulting from the aspiration of the inoculum at the time of inoculation (Fig 1).

**Fig 2:**
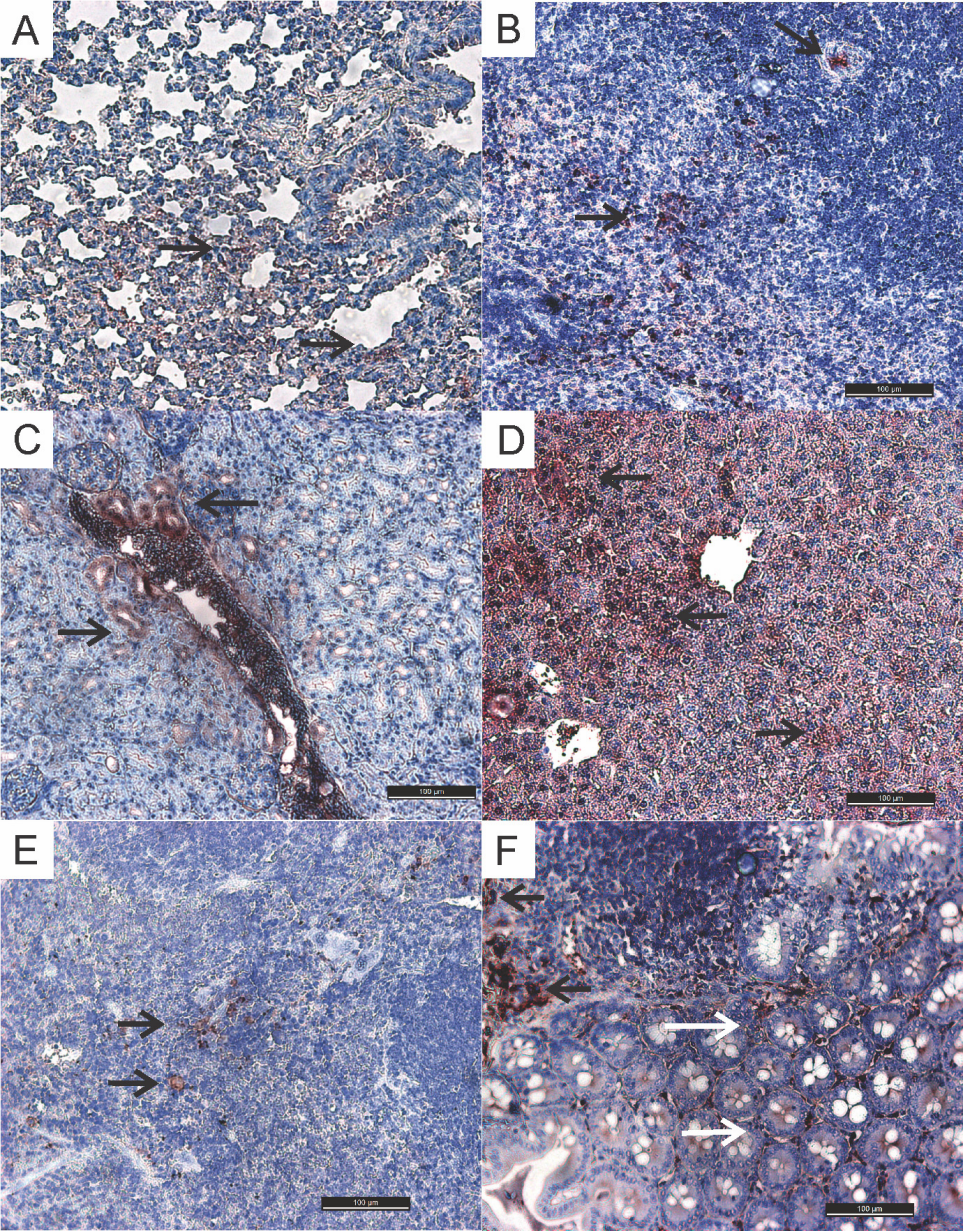
Histological sections of different tissues from mice experimentally infected with VACV-GP2. **Staining using the IHC method (100 μm)**. (A) Lung: mild to moderate immunostaining in the lymphocytes cytoplasms (arrows). (B) Spleen: moderate immunostaining in the lymphocytes cytoplasms (arrows). (C) Kidney: mild-to-moderate immunostaining in the cytoplasm of the proximal-convoluted-tubule epithelial cells (arrows). (D) Liver: mild to moderate immunostaining in the cytoplasm of hepatocytes (arrows). (E) Submandibular lymph nodes: mild immunostaining in the lymphocyte cytoplasm (arrows). (F) Ileum: moderate immunostaining in the cytoplasm of Peyer’s patch lymphocytes (arrows) and mild immunostaining in epithelial cells (white arrow).

Upon IHC, intracytoplasmic immunostainings were observed in several tissues, indicating the presence of the VACV antigen (Fig 2). The immunostainings were observed from the fifth d.p.i. to the 30^th^ d.p.i., except in the lungs, whose specific stainings were detected from the second d.p.i. The intensity and distribution of immunostainings varied between tissues at different times of collection. No intracytoplasmic stainings were observed in the tongue, tonsils, heart, and stomach sections. Focal immunostaining was observed in epithelial cells of the small intestine only at the 20th d.p.i. The detailed description of the immunostainings from each group is outlined in Table 1.

**Table 1:** Immunohistochemistry (IHC) analysis of different tissues of VACV-GP2- infected and no-infected mice.

Time (d.p.i.)	Infected Group
**T2**	Lung (+): pneumocytes and lymphocytes
**T5**	Kidney (+)*: epithelial cells PCT**;
	Spleen (+) to (++): lymphocytes;
	Ileum (+): Peyer’s patch;
	Lung (+): pneumocytes and lymphocytes;
**T10**	Spleen (+) to (++)*:lymphocytes
	Ileum (++): Peyer’s patch;
	Kidney (++): epithelial cells PCT;
	Liver (++): hepatocytes;
	Lung (++): pneumocytes and lymphocytes;
	Mandibular lymph node (++): lymphocytes
**T20**	Liver (++) to (+++): hepatocytes;
	Mandibular lymph node (++): lymphocytes;
	Lung (++): pneumocytes and lymphocytes;
	Spleen (++): lymphocytes;
	Bladder (+): epithelial cells;
	Kidney (+): epithelial cells PCT;
	Ileum (+): epithelial cells and Peyer’s patch
**T30**	Spleen (++): lymphocytes;
	Lung (+): pneumocytes;
	Liver (+): hepatocytes.

*The (+), (++), and (+++) signs classify the VACV-immunostaining as focal, multifocal, and diffused, respectively

**PCT = proximal convoluted tubules

### Plaque-reduction neutralization test (PRNT)

The sera tested using the PRNT method were collected at T0, T10, T20, and T30. Neutralizing antibodies were detected from T20 in 50% of sampled animals, with titers ranging from 1:80 to 1:160.

Table 2 shows all results from feces, blood, OSs, tissues, and serum samples from mice orally inoculated with VACV contaminated milk, analyzed by nested-PCR, IHC, and PRNT methods.

**Table 2:** Analysis of the different samples collected from VACV-GP2–infected mice. Feces, blood, oral swabs (Oss), and tissues were collected at different times, and the nested-PCR, IHC, and PRNT techniques were performed.

Time (d.p.i)	Feces (PCR)	OS (PCR)	Blood (PCR)	Tissues (PCR)	IHC	PRNT
**T1**	-	-	-	-	-	-
**T2**	**N**	**P**	**N**	**Lu, To, To**	**Lu**	**N**
**T3**	-	-	-	-	-	-
**T4**	**N**	**P**	-	-	-	-
**T5**	-	-	**P**	**Ki, He**	**Lu, Ki, Sp, Il**	**N**
**T6**	**P**	**P**	-	-	-	-
**T7**			-	-	-	-
**T8**	**P**	**P**	-	-	-	-
**T9**			-	-	-	-
**T10**	**N**	**P**	**P**	**Ki, Lu, Li, He, To**	**Lu, Ki, Sp, Il, Li, ML**	**N**
**T11**	-	-	-	-	-	-
**T12**	**N**	**N**	**N**	-	-	-
**T13**	-	-	-	-	-	-
**T14**	**N**	**N**	**N**	-	-	-
**T15**				-	-	-
**T16**	**N**	**N**	**N**	-	-	-
**T17**				-	-	-
**T18**	**N**	**N**	**N**	-	-	-
**T19**	-	-	-	-	-	-
**T20**	**N**	**N**	**P**	**Sp**	**Lu, Ki, Sp, Il, ML, Li, Bl**	**NA**
**T21**	-	-	-	-	-	-
**T22**	**N**	**N**	**N**	-	-	-
**T23**	-	-	-	-	-	-
**T24**	**N**	**N**	**N**	-	-	-
**T25**	-	-	-	-	-	-
**T26**	**N**	**N**	**N**	-	-	-
**T27**	-	-	-	-	-	-
**T28**	**N**	**N**	**N**	-	-	-
**T29**	-	-	-	-	-	-
**T30**	**P**	**N**	**P**	**He**	**Lu, SP, Li**	**NA**

P = positive; N = negative; OS = oral swab; Lu = lung; To = tonsil; To = tongue; Ki = Kidney; He = heart; Li = liver; Sp = spleen; ML = mandibular lymph node; Il = ileum; Bl = bladder; NA = neutralizing antibodies. (-) represents the times when there was no samples collection.

## Discussion

This study showed that VACV contaminated milk could be a route of viral transmission to mice, even though no clinical signs were observed in the experimental animals. Previous studies have shown that the murine model is ideal for experiments with VACV and other OPXV, since they are susceptible to the viruses infections, are easily handled and can be used as a model for OPXV transmission studies [10–13; 19–20].

The amount of VACV particles shed in milk of naturally infected cows has not yet been determined, nor is the minimum infectious dose of VACV necessary to infect a human or other animal through consumption of VACV-contaminated milk. However, it is safe to assume that the quantity of VACV detected in milk would be variable, influenced by many factors, such as initial infectious dose, phase of infection, individual variations (i.e. immune response, age, breed). Therefore, the VACV dose used to contaminate the milk in the present study (10^6^ PFU/100 μl) was determined empirically, based in previous studies. Oliveira [6] showed that cows experimentally infected with VACV could eliminate up to 1.8 x 10^7^ genomic units per milliliter (GU/ml) of milk, which corresponds to 10^5^ PFU/ml, approximately. Furthermore, Ferreira and collaborators [10], using 10^7^ PFU/100 μl of VACV-GP2 as inoculum, showed that VACV-experimentally infected mice could shed and horizontally transmitted VACV through feces, using the intranasal route of infection.

VACV-GP2 is a Brazilian strain isolated from cattle [14], which has low virulence in mice, causing a subclinical disease [10]. However, cows intradermally inoculated with VACV-GP2 in the teats had shown clinical disease with typical lesions (vesicles, papules, ulcer, and crust) and systemic dissemination [18; 21–22]. Although in the present study mice exhibited no clinical alterations, the infection was systemic, as shown by the detection of viral DNA in blood, different tissues, feces and saliva. Neutralizing antibodies were detected at the 20^th^ and 30^th^ d.p.i. It is noteworthy that VACV infected rodents captured in BV affected farms did not seemed to have clinical signs and/or any visible lesions that would be associated with VACV infection [3].

The possibility of VACV transmission through milk in humans has not yet been proved. In Brazil, OPXV neutralizing antibodies were detected in human subjects whom had no contact with BV affected cattle and had no clinical signs commonly observed in milkers during BV outbreaks (i.e. exanthematic lesions in the hands, arms, and/or other areas of the body, associated with myalgia, fever and regional lymphangitis) [8]. Moreover, the majority of the subjects of the study (88.8%) had the habit of unpasteurized milk or artisan cheese, made with raw milk, consumption [8]. Although a direct cause-effect relationship could not had been established in that study, those findings suggest that ingestion of VACV contaminated milk and/or dairy products might be a possible route of viral transmission, since VACV subclinical infection seems to be possible in humans with no direct contact with BV affected bovine.

Viral DNA was detected in the feces at the 6^th^, 8^th^, and 30^th^ d.p.i., which suggests that the infection may be persistent, given the long period of shedding. Viral shedding was observed in saliva during the earliest period (from the 2^th^ to the 10^th^ d.p.i.), intermittently in some animals, suggesting that viral DNA may be derived from saliva and not the inoculum. Conversely, DNA in the blood was observed at all collection times, starting at the 5^th^ d.p.i. In a previous study, viral DNA was found in feces and blood from mice inoculated intranasally with VACV-GP2 until the 30^th^ d.p.i [11]. However, shedding through saliva was not observed [11]. VACV-GP2, as previously mentioned, is a strain less virulent to mice. Even though, the results of the present study indicate that virus may have multiplied in the oral mucosa (primary site) following oral inoculation, due to OPXVs tropism for epithelial cells [23], which may explain viral shedding through saliva. Furthermore, dissemination through the lymphatic and/or blood systems might have occurred, as is the case for other routes of inoculation [10–13], followed by shedding through the feces. Studies suggest that VACV fecal shedding in infected animals may have a key role in maintaining and spreading the virus in the environment [10–13,22]. BALB/c mice were infected with VACV following exposure to feces from cattle experimentally infected with VACV-GP2, suggesting that feces from infected cattle may be a source of transmission for rodents [13].

The OPXV *vgf* gene was amplified from spleen, liver, heart, tonsils, tongue, kidney, and lung tissues. These results indicated that milk contaminated with VACV might promote systemic infection in a murine model. Tongue and tonsils are tissues present in the inoculation site and may be the primary sites of viral multiplication. Dissemination to other tissues might have also occurred through the lymphatic system and not only through blood, as previously mentioned. Rivetti and collaborators (2013) have detected VACV DNA in the mesenteric, iliac, and retromammary lymph nodes, as well as the tonsils, spleen and liver of cows experimentally inoculated with VACV-GP2 in the teats. This study showed that the virus has a tropism for lymphoid tissues in cattle [18].

As mice were not perfused prior to the harvest of organs, the detection of VACV DNA in different organs could have been associated with circulating viral particles present in the blood. However, VACV detection in different tissues and organs was also shown upon IHC analysis, indicating that the virus was present in different cells, which suggests that VACV detection by PCR probably was not only due to the presence of VACV-positive blood in the analyzed tissues.

The comparison between the IHC results found in the spleen, liver, kidneys, and lungs were in agreement with the nested-PCR results. However, some tissues tested positive by one method and negative by the other, which might be related to the tissue section and region selected for each method. For example, specific immunostaining in the kidneys and bladder was detected in the IHC until the 20^th^ d.p.i. However, the staining was localized, and the region selected for the PCR might have lacked virus or the viral load was very low, precluding its detection. This finding is important because it suggests that mice inoculated with VACV-GP2–contaminated milk may also shed the virus through urine. Studies in mice and rats infected with *Ectromelia virus* (ECTV) [19–20,24] and *Cowpox virus* (CPXV) [25], respectively, reported the shedding of these viruses through urine, for a long period after inoculation. Viral DNA was detected in the urine of mice nasally inoculated with VACV-GP2 and VACV-WR [11], corroborating this hypothesis.

The ileum is the terminal portion of the small intestine where the Peyer's patches (lymphoid organs) are located. In this study, immunostainings were observed in lymphocytes and macrophages in most ileal sections, and specific staining in epithelial cells only occurred in one section. Previous studies have shown intensive viral multiplication in the gut of mice [11] and cattle [18] infected with OPXV. BALB/c mice nasally infected with VACV-WR and VACV-GP2 strains displayed hyperplasia in Peyer's patches located in the ileum [10–11]. Cattle experimentally inoculated with VACV-GP2 through skin scarification of the teats had systemic dissemination, even in the digestive tract, affecting the mesenteric lymph nodes and the intestine, at the ileal region [18]. Those findings suggest that the lymphatic pathway is more important for viral distribution in those organs, since the immunostaining observed was restricted to such cells.

No specific staining and/or *vgf* gene amplification was observed in the stomach. The stomach of mammals contains a 0.1 M hydrochloric acid (HCl) solution. This extremely acidic medium inactivates many ingested pathogens, including enveloped viruses, as is the case of VACV, and denatures several proteins before their degradation by proteolytic enzymes [26–27]. This emphasizes the hypothesis that a primary site of virus replication after oral inoculation might be the oropharyngeal region.

The lung is an organ irrigated by large-caliber arteries and has lymphoid regions, which would explain the presence of viral proteins in the tissue. All mice showed interstitial pneumonia in the histopathological analyses, including the controls, in a milder form. The pneumonia was most likely caused by aspirating a portion of the inoculum. The presence of inoculum in lungs from groups inoculated with VACV-contaminated milk might have accounted for the early detection of viral proteins and viral DNA by IHC and PCR, respectively. Mice nasally inoculated with VACV-GP2 also showed focal interstitial pneumonia, and immunostainings were observed in the cytoplasm of inflammatory cells from the 1^st^ d.p.i. by IHC. However, no pulmonary lesions were observed in the control group inoculated with PBS [10]. These results were also found in rabbits nasally inoculated with VACV-P1 and VACV-P2 [28]. Milk may trigger a pulmonary inflammatory process when aspirated because it is a fat-, protein-, and carbohydrate-rich substance, which would explain the pneumonia in the animals. However, the pneumonia was more severe in the infected animals, suggesting that this lesion may have been enhanced by viral action.

In the present study, no viable VACV particles were detected in any sample inoculated in BSC-40 cells. Some factors may have contributed to the absence of detectable viable virus particles. First, the amount of each sample was not enough to be tested in duplicate or to be tested again by the different techniques used in this study. Another factor may be related to the inoculation route. Each animal received, orally, milk contaminated with 10^6^ PFU/100μl, and the acid gastric pH may have caused viral degradation, as discussed previously, causing a considerable loss in viral concentration. However, intracytoplasmatic stainings were detected in several tissues using the IHC method, suggesting that viral multiplication occurred in those cells in the different tissues. Similar results were found in another study using VACV-GP2 experimental infection in mice, where no infectious viral particles were detected in tissues from mice nasally inoculated with VACV-GP2. Viable viral particles were detected only in samples from mice inoculated with the most virulent VACV strains [10]. Many studies have shown that mice and other mammals are susceptible to several OPXVs, regardless of the route of inoculation, showing systemic infection and virus shedding through excretions and secretions [10–11; 29–30]. The detection of viral DNA only in feces and saliva, without showing viable viral particles, does not rule out the hypothesis that milk contaminated with VACV may contribute to maintaining and spreading the virus in the environment.

The habit of drinking raw milk, without boiling or pasteurizing it, still can happen in the Brazilian countryside, especially among milkers. Moreover, viable VACV particles have been detected in cheeses made with VACV contaminated milk, at different times of the cheese maturation process [31]. These facts lead to the assumption that VACV oral transmission may occur in humans through the consumption of contaminated milk and/or dairy products. Conversely, preformed lesions present in the oral epithelium may facilitate virus entry, which may lead to the onset of vesicles/ulcers at the site. Thus far, there are two reports of oral and/or oropharyngeal BPXV-related lesions in humans. The authors suggested that the lesions were associated with unpasteurized milk consumption, potentially contaminated with BPXV, which is a subspecies of VACV that occurs mainly in India [9, 32].

This is the first report of the potential risk of VACV-contaminated milk to be a source of viral infection, using an experimental murine model. Furthermore, this study showed that mice are susceptible to this route of transmission, presenting a systemic infection without showing any clinical signs. It was also shown that these animals intermittently shed the virus through feces and saliva, demonstrating that VACV transmission could occur even in the absence of clinical signs. These findings suggest that consumption of VACV contaminated milk and/or dairy products may represent a possible route for human infection, mainly when considering that raw milk and artisan cheeses may be consumed in regions where there is VACV circulation.
